# Genomic analysis of a newly isolated of Japanese encephalitis virus strain, CQ11-66, from a pediatric patient in China

**DOI:** 10.1186/1743-422X-10-101

**Published:** 2013-04-01

**Authors:** Li-Juan Xu, Ruixi Liu, Sheng Ye, Hua Ling, Chao-Min Zhu

**Affiliations:** 1 Department of Infectious diseases and Gastroenterology, Children’s Hospital of Chongqing Medical University, No. 136 Zhongshan Er Road, Yuzhong District, Chongqing 400014, PR China; 2 Ministry of Education Key Laboratory of Child Development and Disorders, No. 136 Zhongshan Er Road, Yuzhong District, Chongqing 400014, China; 3 Key Laboratory of Pediatrics in Chongqing,CSTC2009CA5002), No. 136 Zhongshan Er Road, Yuzhong District, Chongqing 400014, China; 4 Chongqing International Science and Technology Cooperation Center for Child Development and Disorders, No 136 Zhongshan Er Road, Yuzhong District, Chongqing 400014, China; 5 Chongqing Center for Disease Control and Prevention, Chongqing, China

**Keywords:** Japanese encephalitis virus, Genomic analysis, CQ11-66 strain

## Abstract

**Background:**

Japanese encephalitis virus (JEV) is one of the major causative agents of viral encephalitis in East Asia, Southeast Asia and Australia. However, no clinical JEV strain has yet been isolated from JE patients in Chongqing, China. In this study, we report the genomic analysis of a new JEV strain, CQ11-66, isolated from a pediatric patient in Chongqing, China.

**Findings:**

Virus isolation was carried out in BHK-21 cells. Nested PCR was used to detect and isolate the JEV strain, and computer analysis of phylogenetic relationships, nucleic acid homology studies and deduction of the amino acid sequence were conducted using ClustalX (1.8) and Mega5 software. The JEV strain CQ11-66 was isolated from patient cerebrospinal fluid. The sequenced genome of CQ11-66 was 10,863 nucleotides in length, whereas other strains, such as SX09S-01, contain 10,965 nucleotides. Sequence comparison of the CQ11-66 polyprotein open reading frame (ORF) with those of 21 other JEV strains revealed that the nucleotide sequence divergence ranged from 1.68% to 18.46%. Sequence analysis of the full-length CQ11-66 E gene sequence with those of 30 other JEV isolates also identified nucleotide divergence, ranging from 1.69% to 18.74%. Phylogenetic analyses indicated that the CQ11-66 strain belonged to genotype III.

**Conclusions:**

JEV genotype III still circulates in Chongqing and it is therefore important for active surveillance of JEV genotype III to be conducted in the pediatric population.

## Findings

Japanese encephalitis virus (JEV) is the most important causes of epidemic encephalitis in Southeast Asia and Western Pacific regions, with an estimated 35,000 to 50,000 cases each year, and approximately 1-3 of every 1000 JEV infections resulting in severe disease [1]. JEV is a small, enveloped virus with a single-stranded, positive-sense RNA genome of approximately 11,000 nt. Its ORF is translated into a large polyprotein that includes three structural proteins (C, prM and E) and seven non-structural proteins (NS1, NS2A, NS2B, NS3, NS4A, NS4B and NS5) [2]. Since the first JEV isolate was reported in 1949, JE epidemics have occurred in China, and Chongqing is listed as the region of highest incidence. However, no clinical JEV strain has been isolated from human cerebrospinal fluid (CSF), serum or brain tissue of fatal cases in Chongqing; thus, the molecular characteristics and genetic diversity of clinical JEV strains remain unknown.

In this study, acute serum and CSF specimens were collected from patients who were clinically diagnosed with Japanese encephalitis (JE) during July to October in 2011 and hospitalized in the Children’s Hospital of Chongqing Medical University. Serum specimens were diluted in minimal essential medium at a ratio of 1 to 5, pretreated with 100 U ml^-1^ penicillin and streptomycin and placed for at least four hours at 4°C. CSF specimens were incubated directly. A monolayer of BHK-21 cells was grown to 90% confluence and incubated with either CSF or diluted serum at 37°C. Once cytopathic effects (CPE) were observed in a culture, viruses were harvested from the culture medium by centrifugation at 10,000 rpm for 10 min. Viral RNA was extracted from the supernatant (140 μL) of infected BHK-21 cells cultures using the QIAamp® Viral RNA kit (QIAGEN; USA). RNA was reverse-transcribed using a random primer and amplified with a Go Tag® Hot Start Polymerase kit (Promega; USA). The full-length genome was amplified by polymerase chain reaction (PCR) with TaKaRa LA Taq polymerase (TaKaRa; China) using seven pairs of primers (Table 1). The PCR reaction conditions were as follows: 3 min of cDNA denaturation at 94°C, followed by 30 cycles of 94°C denaturation for 30 sec, 55°C primer annealing for 30 sec, and 72°C primer extension for 1 min. Amplified products were examined by agarose gel electrophoresis (1%), purified with a Takara MiniBEST Agarose Gel DNA Extraction Kit Ver 3.0 (TaKaRa; China) and sent to TaKaRa for commercial sequencing. The full-length genome of the strains was compiled using the SeqMan program in the Lasergene software package (DNASTAR). The percentage similarities between aligned nucleotide or amino acid sequences were calculated using MEGA 5 software. Phylogenetic analysis was performed by the neighbor-joining (NJ) method using MEGA5 software. For the previously published JEV strain sequences, the host background, year of isolation, geographic origin and GenBank accession number are listed in Table 2. Multiple sequence alignments and the NJ tree were generated by ClustalX (1.8). The genomic sequence of 21 strains and the E gene sequence of 30 strains were employed in the generation of the phylogenetic trees. The bootstrap probabilities of each node were calculated using 100 replicates.

**Table 1:** Primers for nucleic acid amplification assays

**Name**	**5**^**′**^ -**3**^**′**^	**Length**
PrimerF1	AGTATCGTTGAGAAGAATCG	20
PrimerF2	TATGCTTTCCTGGCGGCGGTAC	22
PrimerF3	GAAGGGGAGACAAGCAGATCAA	22
PrimerF4	CTGCAAGAGAGGAAAAAGACCA	22
PrimerF5	GGAGGGGCAGAGTAGGCAGAAA	22
PrimerF6	CCGTTGGCTCGTGGAGAAAGGA	22
PrimerF7	TCAGCGGAGATGACTGTGTCGT	22
PrimerR1	CCTTCATTTCCTCCTCTTTTGT	22
PrimerR2	TGAACGGCTTTTCCTATGGAGT	22
PrimerR3	CAAACATCAATCCAACTGCCGA	22
PrimerR4	AGTCCATTGGGCATGTGTATGT	22
PrimerR5	TCTGCATGAGCATCGGTTCTTC	22
PrimerR6	AAATGGTTAGAGCAGAAGGGAA	22
PrimerR7	TCTTCCTCACCACCAGCTACAT	22

**Table 2:** Bankground of Japanese encephalitis virus strains used in this study

**Strain**	**Source**	**Genotype**	**Geographical location**	**Year**	**GenBank accession no./reference**	
					**ORF**	**E gene**
Beijing-1	Human	III	China	1948	L48961	L48961
CH1392	Mosquito	III	Taiwan	1990	AF254452	U44960.1
FU	Human	II	Australia	1995	AF217620	L43565.1
GP78	Human	III	India	1978	AF075723	AF075723
SA14-14-2	Vaccin strain	III	China	1954	AF315119.1	AF315119.1
JaGAr01	Mosquito	III	Japan	1959	AF069076	AF069076
WTP	Mosquito	II	Malaysia	1970	HQ223286	U70421
K94P05	Mosquito	I	Korea	1994	AF04555	U34929
JKT5441	Mosquito	II	Indonesia	1981	AF045551	U34929
JKT6468	Mosquito	IV	Indonesia	1981	AY184212	U70407
Ishikawa	Mosquito	I	Japan	1998	AB051292	AB051292
Ling	Human	III	Taiwan	1965	L78128	L78128
RP-9	Mosquito	III	Taiwan	1985	AF014161	AF014161
47	Human	III	China	1950s	AY243827	AY243827
SA14	Mosquito	III	China	1960	U14163	AY243850
K87P39	Mosquito	III	Korea	1987	AY585243	AY585243
P20778	Human	III	India	1958	AF080251	Z34096
CH21955A	Mosquito	III	Taiwan	1994	AF221500	AF221500
ML11	Vaccine	III	Japan	1981	AY508812	U70412
jaOArS982	Mosquito	III	Japan	1982	M18370	M18370
Nakayama	Human	III	Japan	1935	EF571853	AF112297
JKT7003	Mosquito	IV	Indonesia	1981		U70408
Sagiyama	Mosquito	III	Japan	1957		U70419
Kamiyama	Human	III	Japan	1966		AB379813
K82P01	Mosquito	III	Japan	1982		U34926
826309	Human	III	India	1982		Z34094
ThCMAr6793	Mosquito	I	Thaliand	1993		D45363
JKT1724	Mosquito	III	Indonesia	1979		U70404
SH-53	Mosquito	I	China	2001		AY555757
PhAn1242	Pig	III	Philippines	1984		U70417

In total, only one cell culture incubated with a patient CSF sample, named CQ11-66, tested positive by PCR, and none of the cell cultures incubated with patient serum samples tested positive. The genome sequence of CQ11-66 was 10,863 nucleotides in length, with an open reading frame (ORF) (GenBank: KC183732) of 10,296 nt coding for a polyprotein. When comparing its ORF with that of 21 other JEV strains isolated from different geographic regions or periods, high levels of similarity were observed, with nucleotide divergence ranging from 1.68% to 18.46%, and amino acid divergence ranging from 0.32% to 4.89%. A comparison of the CQ11-66 polyprotein ORF with that of SA14-14-2, the attenuated JEV vaccine strain currently widely used in China, discovered a total of 201 nucleotide differences (1.96%) and 42 amino acid differences (1.23%).

**Figure 1:**
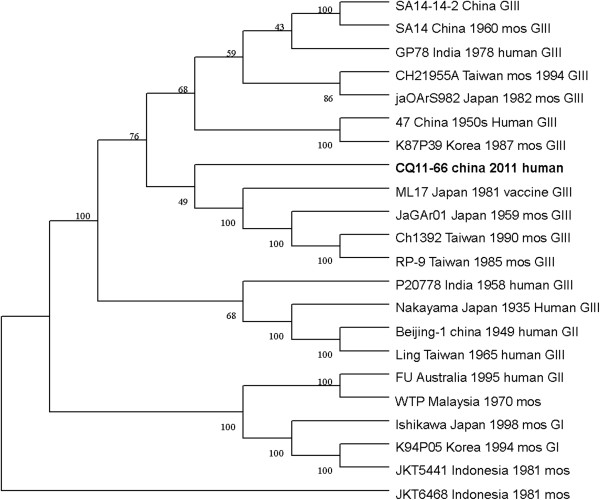
**Phylogenetic tree constructed by the neighbor-joining method, based on the polyprotein ORFs of selected Japanese encephalitis virus strains.** The CQ11-66 China 2011 human obtained in this study is shown in bold face.

**Figure 2:**
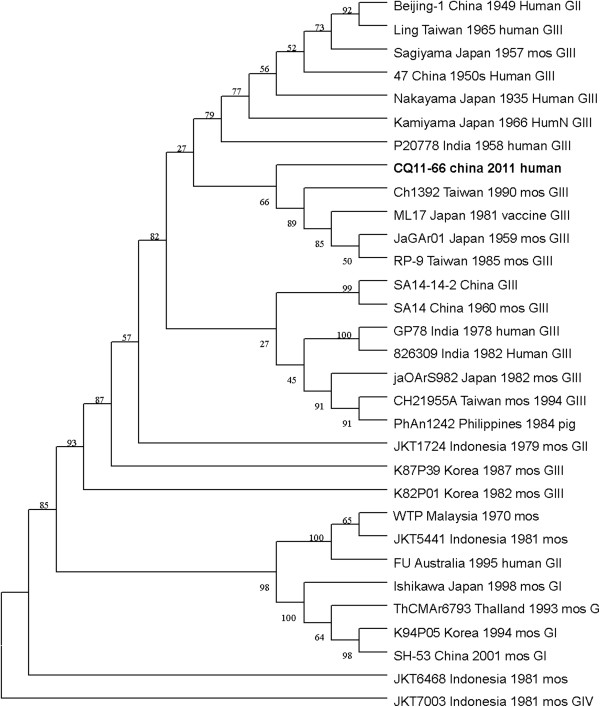
**Phylogenetic tree constructed by the neighbor-joining method, based on the envelope (E) protein gene of selected Japanese encephalitis virus strains.** The CQ11-66 China 2011 human obtained in this study is shown in bold face.

The nucleotide and amino acid sequences of the CQ11-66 strain E region were compared with those of other isolates available in GenBank. This analysis revealed that nucleotide divergence ranged from 1.62% to 18.74%, while deduced amino acid divergence ranged from 1.01% to 5.13%. Among these amino acid mutations, the E408 (Arg to Lys) variation was unique to CQ11-661. A comparison of the CQ11-66 strain E gene with that of SA14-14-2 also indicated 36 nucleotide changes, resulting in 15 amino acid changes: E76 (Thr to Met), E107 (Phe to Leu), E138 (Lys to Glu), E176 (Val to Thr), E177 (Ala to Thr), E209 (Lys to Arg), E227 (Ser to Pro), E244 (Gly to Glu), E264 (His to Gln), E279 (Met to Lys), E306 (Glu to Gly), E315 (Val to Ala), E408 (Arg to Lys), E416 (Asp to Gly) and E439 (Asp to Gly). On the basis of the ORF gene sequences of 22 JEV isolates and the E gene sequences of 31 JEV isolates, phylogenetic trees were constructed (shown in Figures 1 and 2). Both phylogenetic trees provided similar topology, and the analysis indicated that the CQ11-66 strain belonged to genotype III.

On the basis of 240 nucleotide of the C/prM gene, JEV strains were divided into five genotypes two decades ago [3,4]. However, the functional significance of the prM genetic variations remains to be established, and genetic relationships based on short sequences (<300 nt) should be required to take into account the biological significance and may be considered with caution [5,6]. Subsequently, the full-length E gene of JEV and its deduced protein sequence have been used as reliable phylogenetic markers, given that the E protein plays an important role in the JEV life cycle [7]. In China, many JEV strains belonging to genotype I and genotype III have been reported, and it was suggested by surveillance data that genotype I strains have been gradually replacing genotype III as the dominant strains in Asia. Due to limited sequence information regarding JEV isolates in Chongqing, the dominant genotype in one of the high-incidence regions in China remains unclear. In this study, phylogenetic analyses employing the ORF and E gene sequence of the CQ11-66 strain indicated it belonged to genotype III.

The crystallographic structure of the E protein of the flavivirus tick-borne encephalitis virus revealed that the E protein consists of three domains. The difference in the E gene of JEV strains, particularly in domain III [8], may contribute to the diversity of neutralizing and antigenic properties [9]. Previous research has also indicated that mutations in domain III modulated virus binding and entry into host cells [10,11]. In this study, comparison of the E gene of CQ11-66 strain with the vaccine strain SA14-14-2 revealed 15 amino acid substitutions. Among these variations, the Glu to Lys alteration at position 138 is consistent with previous observations that the E138 mutation is associated with attenuation of the JEV strain and inhibits viral spread from cell to cell [12]. Other groups have also demonstrated that mutations of residues E49, E138, E306 and E389 of the E protein reduce the efficiency of viral binding to heparan sulphate residues on target cells [13]. In our results, a substitution (Met to Lys) was observed at the position E279, which may increase virulence in mice [14].

Genetic variation among JEV strains isolated from widely different time periods and geographical regions has been reported in many studies [13]. A previous study examining 46 JEV strains found that those from a much wider geographic region and at certain time period were similar, but that genetic variation was present among strains from diverse regions or from different time periods in the same region. These observations suggested JEV is continuously evolving [15]. Meanwhile, Yun et al. [16] reported that the E gene sequences of the 10 Korean JEV strains, despite differences in their geographic distributions and the maximum five-year time span, showed remarkable genetic stability. Such stability in the JEV genome was also observed in strains from China and Japan [17,18]. In this study, phylogenetic analyses of the CQ11-66 ORF and the ORF of 21 other JEV strains isolated from several countries revealed the lowest nucleotide divergence and the lowest amino acid divergence with the JaGA01 strain. Meanwhile, phylogenetic analyses of its E gene and the E gene of 30 other JEV strains revealed the lowest nucleotide divergence and the lowest amino acid divergence with the JaGA01 strain.

In conclusion, we have isolated a JEV strain from a JE patient in Chongqing, China. Molecular analysis of the nucleotide and amino acid sequences indicated that CQ11-66 belonged to genotype III, and comparison of the ORF sequence and the E gene sequence with other strains from different regions at different periods revealed high homology.

### Ethics statement

The study was performed after consultation with the patients or their guardians and after the receipt of written consent. The study-related information was used anonymously. The Institutional Review Board of the Children’s hospital of Chongqing Medical University approved the research involving human materials.

## Competing interests

The authors declare that they have no competing interests.

## Authors’ contributions

LJX performed the experiments, prepared of the manuscript, collected the specimens and contributed to the data analysis. SY contributed to the preparation of the manuscript. CMZ and HL designed the study. All authors have read and approve the final manuscript.
